# Sustainable Recovery of Polyphenols and Carotenoids from Horned Melon Peel via Cloud Point Extraction

**DOI:** 10.3390/foods13182863

**Published:** 2024-09-10

**Authors:** Vanja Travičić, Teodora Cvanić, Senka Vidović, Lato Pezo, Alyssa Hidalgo, Olja Šovljanski, Gordana Ćetković

**Affiliations:** 1 Faculty of Technology Novi Sad, University of Novi Sad, Bulevar cara Lazara 1, 21000 Novi Sad, Serbia; teodora.cvanic@uns.ac.rs (T.C.); senka.vidovic@uns.ac.rs (S.V.); oljasovljanski@uns.ac.rs (O.Š.); gcetkovic@uns.ac.rs (G.Ć.); 2 Engineering Department, Institute of General and Physical Chemistry, Studentski trg 12/V, 11000 Belgrade, Serbia; latopezo@yahoo.co.uk; 3 Department of Food, Environmental and Nutritional Sciences (DeFENS), Università degli Studi di Milano, Via Celoria 2, 20133 Milan, Italy; alyssa.hidalgovidal@unimi.it

**Keywords:** horned melon peel, polyphenols, carotenoids, cloud point extraction, antioxidant activity

## Abstract

Using natural plant extracts as food additives is a promising approach for improving food products’ quality, nutritional value, and safety, offering advantages for both consumers and the environment. Therefore, the main goal of this study was to develop a sustainable method for extracting polyphenols and carotenoids from horned melon peel using the cloud point extraction (CPE) technique, intending to utilize it as a natural food additive. CPE is novel promising extraction method for separation and pre-concentration of different compounds while being simple, inexpensive, and low-toxic. Three parameters within the CPE approach, i.e., pH, equilibrium temperature, and equilibrium time, were investigated as independent variables through the implementation of Box–Behnken design and statistical analyses. The optimized conditions for the maximum recovery of both polyphenols and carotenoids, reaching 236.14 mg GAE/100 g and 13.80 mg β carotene/100 g, respectively, were a pH value of 7.32, an equilibrium temperature of 55 °C, and an equilibrium time of 43.03 min. The obtained bioactives’ recovery values under the optimized conditions corresponded to the predicted ones, indicating the suitability of the employed RSM model. These results highlight the effectiveness of CPE in extracting bioactive compounds with varying polarities from agricultural by-products, underscoring its potential for enhancing the value of food waste and advancing sustainable practices in food processing. According to microbiological food safety parameters, the optimal CPE extract is suitable for food applications, while its storage under refrigerated and dark conditions is particularly beneficial. The CPE extract’s enhanced stability under these conditions makes it a more viable option for long-term storage, preserving both safety and quality.

## 1. Introduction

Food waste is a significant global issue affecting the entire supply chain. Due to spoilage, approximately 1.3 billion metric tons of foods are wasted annually [1]. According to the Food and Agriculture Organization of the United Nations (FAO), fruits and vegetables have significantly higher wastage rates compared with other food groups, estimated at approximately 195 million metric tons annually [2]. Apart from causing hunger, food insecurity, and economic losses, food waste also has a notable environmental impact. Its decomposition in landfills produces methane, a powerful greenhouse gas that significantly contributes to climate change.

Horned melon, also known as kiwano (*Cucumis metuliferus* E. Mey. Ex. Naudin), belongs to the Cucurbitaceae family and serves as a rich source of various nutritional and phytochemical components crucial in the human diet [3]. The fruit boasts ripe green flesh filled with white seeds, encased in an orange-yellow rind adorned with spiny outgrowths. The flesh, when ripe, is commonly consumed raw or processed into refreshing fruit juice [4]. Importantly, horned melon possesses valuable health benefits as it has antioxidant, antidiabetic, antifungal, and antibacterial effects due to the presence of compounds like phenolics, flavonoids, glycosides, and carotenoids, which has led to the increasing popularity and consumption of this fruit. While every part of the horned melon is edible, it is typical for most people to only eat the inner portions, discarding the remainder as waste. The increased production of this fruit has led to the generation of substantial quantities of non-edible components, such as peels and seeds, which have been observed to be a renewable source of valuable bioactive compounds [5]. Utilizing these by-products not only provides an eco-friendly waste management solution but also offers promising opportunities for integration into the food industry [6,7,8]. Currently, there remains limited information concerning the potential uses of horned melon peel. Specifically, horned melon peel can be processed into extracts rich in antioxidants and dietary fiber, which can be incorporated into food products such as natural food colorings, flavorings, or dietary supplements. This utilization can enhance the nutritional profile of food items but also add a unique tangy flavor, contributing to the diversification and innovation within the food sector.

The food industry is shifting towards environmentally friendly practices for extracting food bioactives, driven by the limitations of traditional extraction methods in terms of technology, science, ecology, and economy. Traditional extraction methods often rely on organic solvents such as ethanol or hexane, which can have environmental and health impacts due to their potential toxicity and flammability. Additionally, these methods can be time-consuming and require large amounts of energy for solvent recovery and purification processes. The use of organic solvents also raises concerns about residual solvent levels in the extracted bioactive compounds, which may affect their safety and suitability for use in food products. Eco-friendly extraction methods, often referred to as “green methods”, play a crucial role in addressing environmental and health-related issues. Eco-friendly extraction of food bioactive compounds aims to develop processes that minimize energy usage and eliminate the need for organic solvents while ensuring the safety and quality of the extracted compounds [9]. Innovative techniques such as supercritical fluid extraction, ultrasound-assisted extraction, microwave-assisted extraction, and high hydrostatic pressure are being employed to improve efficiency and reduce environmental impacts [10,11]. These eco-friendly extractions result in higher yields and reduced solvent consumption, meeting the criteria for simplicity, speed, and cost effectiveness, thereby balancing economic feasibility with environmental stewardship.

According to Travičić et al. [12], cloud point extraction (CPE), also known as surfactant-based extraction, liquid-concentration technique, micelle extraction, or micelle-mediated extraction, is a versatile method that offers several advantages over traditional extraction methods. These advantages include increased selectivity, reduced solvent consumption, lower energy requirements, and improved compatibility with sensitive compounds. Additionally, CPE enables the extraction of both hydrophilic and hydrophobic compounds, making it a versatile and efficient technique for extracting bioactive compounds from various sources. Additionally, CPE is typically performed at mild or low temperatures without the use of hazardous or toxic reagents. It does not require special equipment, making it a cost-effective and straightforward method to implement. One of the main advantages of CPE is the use of primarily food-grade surfactants, ensuring its safety for food-related applications [13].

During the CPE process, a non-ionic surfactant induces the formation of hydrophobic micelles, which then entrap hydrophobic bioactive compounds, protecting them from environmental factors and potential alterations such as oxidation [14,15]. Under suitable conditions, extraction occurs at the cloud point temperature, where the surfactant becomes cloudy—less soluble or even insoluble compared with the initial sample—leading to phase separation into a surfactant-rich phase and an aqueous phase. This process results in the preconcentration of analytes such as carotenoids and phenols within the surfactant-rich phase, due to the clouding phenomenon [16]. In previous studies regarding the isolation of phenolic compounds [17,18,19,20,21,22], CPE was applied using surfactants such as Triton X-114, Brij S20, Tween 20 and 80, PEG-8000, Span 20, and lecithin. Meanwhile, for the isolation of carotenoids [23,24] CPE was applied using lecithin and Tween 20. According to the U.S. Food and Drug Administration (FDA), these surfactants are considered edible.

To the best of our knowledge, there are no studies concerning the extraction of polyphenols and carotenoids (as bioactives with different polarities) from horned melon peel using CPE. The primary objective of this research was to optimize cloud point extraction (CPE) parameters to obtain extracts rich in bioactive compounds, particularly carotenoids and phenolics, with high antioxidant potential in the surfactant-rich CPE phase, suitable for applications as food additives. To achieve this goal, this study employed a response surface methodology (RSM) approach to improve model fitting quality, analyze key factors affecting the extraction process, and enhance the accuracy of extraction optimization. Water CPE phases were tested in parallel to evaluate all potential residues of phenols and antioxidant potential. Finally, microbiological food safety parameters as well as storage stability of obtained extracts related to the microbiological parameters were investigated.

## 2. Materials and Methods

### 2.1. Plant Material and Other Chemicals

Horned melon fruits were sourced from an organic agricultural holding situated in the Fruška Gora Mountain region on the Pannonian plain near Novi Sad, Republic of Serbia (latitude 45°12′ N, longitude 19°45′ E). The peel of the horned melons was manually separated under sterile laboratory conditions and stored after freeze drying. The main drying process was performed in a Martin Crist Alpha 2–4 (Osterode, Germany) freeze dryer at a pressure of 0.01 mbar and temperatures from −40 to 20 °C for 48 h. Subsequently, a final drying phase lasting 4 h at a pressure of 0.005 mbar and temperatures between 20 to 30 °C was conducted. The collected freeze-dried horned melon peel was then pulverized using a laboratory grinder (B800E high-speed grinder, Gorenje, Velenje, Slovenia). After grinding, the average particle size of dried powdered material was determined using a sieve set (CISA Cedaceria Industrial, Barcelona, Spain) and calculated as 71.5 µm. Obtained plant material was stored at freezing temperatures before further use.

Tween 80 was obtained from Alfa Aesar (Karlsruhe, Germany). Folin–Ciocalteu reagent, 2,2-diphenyl-1-picrylhydrazyl radical (DPPH•), and Trolox were purchased from Sigma Chemical Co. (St. Louis, MO, USA). All other chemicals and solvents used for assays were of the highest analytical grade. Ultrapure water was produced using an Elix_3 Millipore water purification system coupled to a Milli-Q module (model Advantage10) (Millipore, Molsheim, France).

### 2.2. Horned Melon Peel Extraction

#### 2.2.1. Cloud Point Extraction of Horned Melon Peel

Three parameters—pH (1.5–7.5), equilibrium temperature (35–75 °C), and equilibrium time (20–60 min)—were investigated as independent variables in the CPE approach, while all other parameters were kept constant. For each experimental run, horned melon peel powder, water, and Tween 80 were added to a 100 mL conical flask with a solid-to-liquid ratio of 1:70 (*w*/*v*) and a surfactant concentration of 10% (*w*/*v*). The pH of the solution varied between 1.5 and 7.5. The mixture was thoroughly stirred for 20 min on a magnetic stirrer at 45 °C, followed by centrifugation (model: EBA 21, Hettich Zentrifugen, Tuttlingen, Germany) at 4000 rpm for 10 min to separate the supernatant. The supernatant was then mixed with NaCl at a concentration of 18% (*w*/*v*). The mixture was incubated in a temperature-controlled water bath (model: VIMS elektrik, Loznica, Tršić, Serbia) at varying temperatures (35–75 °C) and times (20–60 min), followed by centrifugation to achieve phase separation (4000 rpm for 10 min). The bottom water phase was removed using a pipette, and the remaining surfactant phase was used to estimate total carotenoids (TCs), total phenols (TPs), and antioxidant activity (AA).

#### 2.2.2. Conventional Water Extraction of Horned Melon Peel

Horned melon peel powder (1 g) was extracted in 70 mL distilled water for 3 h on a magnetic stirrer at 40 °C. The liquid extract was separated by centrifugation at 4000 rpm for 10 min. The supernatant was analyzed for TCs, TPs, and AA.

### 2.3. Analytical Methods

#### 2.3.1. Determination of Total Carotenoids Content (TC_sp_)

Total carotenoid content (TC_sp_) in the surfactant-rich phase of the extract was determined spectrophotometrically using the method described by Nagata and Yamashita [25]. Briefly, 250 µL of extract was added to each microplate well, while extracting solvent was used as the blank. The content of total carotenoids was calculated using the equation:

TC_sp_ (mg β-carotene/100 mL) = 0.216·A_663_ − 1.22·A_645_ − 0.304·A_505_ + 0.452·A_453_ (1)

where A_663_, A_645_, A_505_, and A_453_ represent the absorbances measured at 663, 645, 505, and 453 nm, respectively. The total carotenoid content was expressed as mg of β-carotene equivalents per 100 g of sample.

#### 2.3.2. Determination of Total Phenols Content (TP_sp_ and TP_wp_)

Total phenols content (TP_sp_—total phenolics content in the surfactant-rich phase and TP_wp_—total phenolics content in the water phase) of the extracts was established using the Folin–Ciocalteau spectrophotometric method adapted to microscale [26]. Each microplate well contained a mixture of 15 μL of sample, 170 μL of distilled water, 12 μL of the Folin–Ciocalteu reagent, and 30 μL of 20% (*w*/*v*) sodium carbonate. The prepared microplate was incubated for 1 h and the absorbances were measured at 750 nm. Distilled water was used as blank. The obtained results were expressed as mg gallic acid equivalents (GAE) per 100 g of sample.

#### 2.3.3. Determination of DPPH Radical Scavenging Activity (DPPH_sp_ and DPPH_wp_)

The DPPH radical scavenging assay (DPPH_sp_—DPPH radical scavenging activity in the surfactant-rich phase and DPPH_wp_—DPPH radical scavenging activity in the water phase) was performed using spectrophotometrically-based method [5]. In brief, 250 μL DPPH● solution in methanol (0.89 mM) was mixed with 10 μL of sample in a microplate well. Absorbance was measured at 515 nm after 50 min incubation in the dark at ambient temperature. Methanol was used as blank. The results were expressed in μmol Trolox equivalent (TE) per 100 g of sample.

#### 2.3.4. Determination of Reducing Power (RP_sp_ and RP_wp_)

Reducing power (RP_sp_—reducing power in the surfactant-rich phase and RP_wp_—reducing power in the water phase) was determined by Oyaizu’s method [27] modified for a 96-well microplate. In brief, 25 μL of sample extract or 25 μL water (blank test), 25 μL sodium phosphate buffer (pH = 6.6), and 25 μL of 1% potassium iron(III) cyanide were mixed and incubated in a water bath for 20 min at 50 °C. After cooling, 25 μL of 10% trichloroacetic acid was added and solutions were centrifuged at 4000 rpm for 10 min. After centrifugation, 50 μL of supernatant was mixed with 50 μL of distilled water and 10 μL of 0.1% iron(III) chloride in the microplate well. Absorbance was measured immediately at 700 nm. The results were expressed as μmol Trolox equivalent (TE) per 100 g of sample.

#### 2.3.5. Determination of ABTS Radical Scavenging Activity (ABTS_sp_ and ABTS_wp_)

The ABTS radical scavenging assay (ABTS_sp_—ABTS radical scavenging assay in the surfactant-rich phase and ABTS_wp_—ABTS radical scavenging assay in the water phase) was conducted using a modified procedure based on that described by Tumbas Šaponjac et al. [28]. The absorbance of 250 μL activated ABTS+● (with MnO_2_) before and 35 min (incubated at 25 °C) after the addition of 2 μL of sample extract was measured at 414 nm. Water was used as blank. The results were expressed as μmol Trolox equivalent (TE) per 100 g of sample.

### 2.4. Experimental Design

To extract bioactive compounds with varying polarities from horned melon peel, a response surface methodology (RSM) experimental design was employed to evaluate the effects of CPE parameters and optimize conditions for multiple responses. The Box–Behnken design (BBD) was used, involving three variables at three levels. The design included fifteen randomized runs with three replicates at the central point. The independent variables were pH (X_1_), equilibrium temperature (X_2_), and equilibrium time (X_3_), coded from −1 to 1, making the units of the variables irrelevant (Table 1). The actual values were selected based on preliminary experimental results. Optimal extraction conditions were determined by considering responses such as total carotenoid content, total phenols content, DPPH radical scavenging activity, reducing power, and ABTS radical scavenging activity.

### 2.5. Evaluation of Selected Food-Related Parameters

The microbiological safety of raw materials used for extract preparation (water, NaCl, Tween 80, lyophilized horned melon peel) as well as water-based and CPE extracts was assessed by evaluating various microbiological parameters using standard microbiological methods. The parameters included total viable count, *Escherichia coli*, *Salmonella* spp., *Staphylococcus aureus*, *Listeria monocytogenes*, yeast and mold counts, *Bacillus cereus*, and Enterobacteriaceae. For the total viable count, samples were plated on nutrient agar and incubated at 37 °C for 48 h. To detect *Escherichia coli*, samples were plated on TBX agar and incubated at 44 °C for 24 h. For *Salmonella* spp. and *Listeria monocytogenes*, samples were enriched in selective broths (selenite F broth for *Salmonella*, listeria enrichment broth for *Listeria*), followed by plating on selective agars (XLD for *Salmonella*, PALCAM agar for *Listeria*) and incubated at 37 °C for 24–48 h. *Staphylococcus aureus was* detected by plating samples on Baird–Parker agar and incubating at 37 °C for 24–48 h; black colonies with clear zones indicated *S. aureus* presence. Yeast and mold counts were determined by plating samples on Sabouraud dextrose agar (SDA) and incubating at 25 °C for 5 days. *Bacillus cereus* detection involved plating samples on Mannitol egg yolk polymyxin (MYP) agar and incubating at 30 °C for 24 h, with pink colonies and zones of precipitation indicating *B. cereus*. Enterobacteriaceae were detected by plating samples on violet red bile glucose (VRBG) agar and incubating at 37 °C for 24 h, with red colonies indicating their presence. All nutrient media and raw materials for microbiological analysis were obtained from HiMedia (Mumbai, India). The microbiological data were analyzed to determine the effectiveness of the CPE process in comparison to another extraction method.

Storage stability testing was used for the evaluation of the stability of the CPE extract. Both the water-based and CPE extracts were stored under various conditions for 60 days, including room temperature with light (RT+L), room temperature without light (RT−L), refrigerated with light (FT+L), and refrigerated without light (FT−L). Extracts were aliquoted into sterile containers and stored under the specified conditions. Samples were taken at 0, 3, 4, 14, 21, 30, and 60 days for analysis. Total viable count and yeast and mold counts were measured at each time point using the methods described above. Changes in microbial counts were recorded and compared with the initial values.

### 2.6. Statistical Analysis

#### 2.6.1. Principal Component Analysis

Principal component analysis (PCA) was used to explain and identify patterns within the collected data relating to the cloud point extraction of bioactive components from horned melon peel.

#### 2.6.2. Response Surface Methodology

The cloud point extraction process to obtain bioactive components from horned melon peel was influenced by three-factor variables—pH (X_1_) of 1.5, 4.5, and 7.5; equilibrium temperature (X_2_) of 35, 55, and 75 °C; and equilibrium time (X_3_) of 20, 40, and 60 min—as detailed in the experimental plan in Table 1. These factor ranges were established based on preliminary trials. Utilizing a design with three levels for each of the three factors, the experimental data were analyzed to assess the effects. A sample size of 15 was deemed sufficient for evaluating second-order polynomial (SOP) coefficients [29]. For visualization of the obtained results, contour plots were constructed for the interaction of the X_2_ and X_3_ variables, while X_1_ was held at the center point. Analysis of Box–Behnken design data was conducted via Minitab Statistical Software 22 using response surface regression, contour plot, and response optimization functions. Additionally, analysis of variance (ANOVA) was conducted to specifically evaluate the impact of factor variables on the responses. These ANOVA calculations were performed using TIBCO Statistica^®^ 14.0.0.15 software [STATISTICA (Data Analysis Software System) V14.0.0.15; TIBCO Stat-Soft Inc.: Tulsa, OK, USA, 2020].

#### 2.6.3. Standard Scores

Standard scores were evaluated across the trials conducted using the cloud point extraction process. The ranking method was based on the ratio between the raw data and the extreme values for each parameter and response [30]. The higher pH value and the lower equilibrium temperature and equilibrium time were considered favorable.

## 3. Results and Discussion

Surfactant-based aqueous solutions have emerged as a promising method for enhancing the extraction of biologically active compounds from plant materials. Previous research has demonstrated their effectiveness. For instance, Motikar et al. [18] highlighted the benefits of various techniques such as CPE, ultrasound-assisted CPE, and microwave-assisted CPE for extracting polyphenols from pomegranate peel, showing superior efficiency compared with traditional extraction methods. Additionally, Giovanoudis et al. [21] investigated the influence of different solvents (water, ethanol, 60% ethanol, and the surfactant Tween 80 used in CPE) on polyphenol recovery. They found that the CPE method achieved the highest recovery of total polyphenol content, surpassing water, ethanol, and 60% ethanol extractions by 36.89%, 61.50%, and 20.06%, respectively. Takla et al. [31] also confirmed the significant efficiency of the CPE approach over conventional methods for extracting various bioactive alkaloids. Despite the many advantages, it can still be said that there is a lack of information on the extraction of bioactives from plant materials using CPE. Therefore, this study aimed to fill the current gap in the knowledge by investigating and optimizing CPE parameters for the simultaneous extraction of bioactive compounds with different polarities from horned melon peels.

Experimental results of total carotenoids (TCs), total phenols (TPs), DPPH radical scavenging activity (DPPH), reducing power (RP), and ABTS radical scavenging activity (ABTS) obtained under different CPE conditions are presented in Table 2. For a comprehensive evaluation, both obtained phases, the surfactant-rich phase and the water phase, were tested.

**Figure 1:**
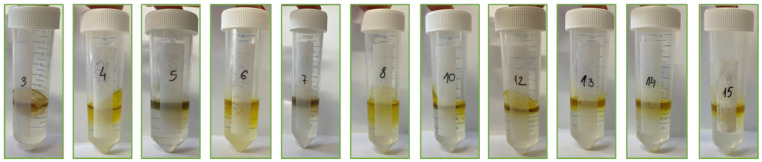
Appearances of the CPE extracts of horned melon peel obtained according to BBD experimental design.

Figure 1 shows the appearance of the CPE extracts from horned melon peel obtained using the BBD experimental design. It was observed that the carotenoids were concentrated in the surfactant-rich phase, so their concentration in the water phase was not measured.

The extraction yield of carotenoids was notably influenced by pH levels. At a more acidic pH (1.5), the lowest TC value was recorded; sample 3 contained 1.26 mg β-carotene/100 g and samples 5 and 7 contained 1.87 mg β-carotene/100 g. The highest yields of TCs were observed at pH 7.5 in samples 2, 4, and 6, ranging from 12.55 to 14.65 mg β-carotene/100 g. Low pH can cause the breakdown of the conjugated double-bond system, which gives carotenoids their bright colors, leading to the formation of colorless or differently colored compounds and resulting in a darkened extract [32]. Acidic conditions also accelerate the oxidation of carotenoids and xanthophylls, resulting in brown-colored byproducts [33]. Additionally, acidic conditions can promote the formation of complexes between carotenoids, xanthophylls, and other compounds such as metals or polyphenols, which can exhibit different colors than the free carotenoids and xanthophylls, potentially resulting in a darker extract [34].

In terms of polyphenol extraction yield, equilibrium temperature and equilibrium time were more significant factors than pH. At a temperature of 35 °C, micellar and aqueous phase separation did not occur. In general, CPE is achieved when the equilibrium temperature of the extraction solution is higher than the cloud point temperature (CPT) of the surfactant [35]. At temperatures below the CPT, surfactants remain homogeneously distributed in the solution and do not form micellar aggregates sufficiently to cause phase separation. At 35 °C, which was probably below the CPT, surfactants did not organize into the micellar structures necessary for phase separation, resulting in the absence of a micellar phase distinct from the aqueous phase. To achieve an efficient CPE process, it is essential to raise the solution temperature above the cloud point. At higher temperatures, the interactions between surfactants and water decrease, enabling the surfactants to aggregate and form a separate micellar phase. Therefore, to effectively achieve CPE, careful control of the extraction system’s temperature is required to ensure adequate phase separation and optimize the extraction of the desired bioactive compounds.

In the surfactant-rich phase, TP_sf_ values were in a range from 62.33 to 277.34 mg GAE/100 g. The highest yields were noted for sample 8 (pH 7.5; 55 °C; 60 min) and sample 6 (pH 7.5; 55 °C; 20 min. However, when experiments were conducted at 75 °C, a decrease in TP was observed. This decrease aligns with findings by Motikar et al. [18], who also noted reduced yields of polyphenols and flavonoids at temperatures higher than 55 °C. This decline in polyphenol content at higher temperatures may be attributed to thermal degradation or changes in surfactant properties, potentially leading to decreased solubility or stability of polyphenols.

Increasing the extraction time from 20 to 65 min led to a rise in the levels of TP_sf_ and TC_sf_ in samples 8 and 6, although there were no notable differences in TC_sf_. This extraction time range aligns with findings from Skrypnik and Novikova’s [36] study, where they optimized the extraction of polyphenols from apple pomace using nonionic emulsifiers. Vieira and Ventura [23] reported a higher optimal extraction time (140 min) using aqueous solutions of Tween 20 for the extraction of carotenoids from microalgae *Saragassym muticum*. However, they did not include the salting-out effect in their protocol. Typically, leveraging the salting-out phenomenon in CPE reduces the time required for separation. This probably explains the shorter extraction time observed in our study for effectively extracting carotenoids.

Determining the efficacy of extracting compounds from a sample depends on the isolated compounds’ inherent traits. Hence, it was crucial to assess the antioxidant properties of the extracted bioactives. To achieve this, three antioxidant assays were employed. According to Table 2, both inhibition of and an increase in antioxidant activity were observed. A similar behavior of the reaction mixture with Tween 80 was observed by Skrypnik and Novikova. Previous studies have indicated that surfactants in the reaction mixture can influence both the antioxidant properties of bioactive compounds and the reaction mechanism with free radicals [36,37,38]. Conversely, Pérez-Rosés et al. [39] demonstrated that Tween 20 and Tween 80 did not exhibit scavenging activity for DPPH radicals and their presence in the medium did not interfere with the analysis of antioxidant activity. Non-ionic surfactants may bind to isolated bioactive compounds, altering their structural configuration or impeding their interaction with free radicals, thereby diminishing their efficacy. Additionally, the formation of micelles facilitated by non-ionic surfactants can encapsulate antioxidant molecules, restricting their accessibility for scavenging free radicals and consequently reducing overall antioxidant activity. Lastly, structural modifications to antioxidant molecules caused by non-ionic surfactants can impair their capacity to donate hydrogen atoms or electrons, obstructing their antioxidant function. To address this issue more accurately, further studies are needed to investigate not only the impact of Tween 80 on the yield of polyphenols and carotenoids, but also on its effect on the antioxidant properties of the recovered compounds.

Considering the high efficiency of carotenoid recovery via single-step CPE, the additional step of CPE was not applied in this study. However, to examine the residues of the second group of target compounds (polyphenols) in the water phase, an analysis of their content and antioxidant activity was performed. Based on the results in Table 2, it was concluded that a significant concentration of polyphenols remained in the aqueous phase. In general, polyphenols are characterized by their structural diversity and hydrophilic nature, and additional CPE steps to achieve optimal recovery are often required [40]. This also includes further optimization of conditions such as pH, temperature, and surfactant concentration across multiple steps. The potential of double-step CPE for polyphenol recovery using Tween 80 was confirmed by Giovanoudis et al. [41].

Table 3 presents a correlation matrix of various cloud point extraction parameters. The relationships between different measurements in the surfactant-rich phase (sp) and water phase (wp) were evaluated. The parameters included total carotenoids content (TCs), total phenols content (TPs), radical scavenging activity (DPPH), reducing power (RP), and ABTS radical scavenging activity (ABTS). Based on the correlation analysis, several significant relationships were observed. TCs exhibited a negative correlation with DPPH_sp_ at a highly significant level (*p* < 0.001). Additionally, TCs were negatively correlated with TP_wp_ and DPPH_wp_ at a significance level of *p* < 0.05. TP_wp_ demonstrated a significant positive correlation with DPPH_sp_ at a level of *p* < 0.01. Furthermore, TP_wp_ showed positive correlations with DPPH_wp_ and RP_wp_ at a significance level of *p* < 0.05. Conversely, TP_wp_ was negatively correlated with ABTS_sp_ at a significance level of *p* < 0.05. DPPH_sp_ displayed a notable positive correlation with DPPH_wp_ at a level of *p* < 0.01. Conversely, DPPH_sp_ exhibited a negative correlation with RP_wp_ at a significance level of *p* < 0.05. DPPH_wp_ was negatively correlated with ABTS_sp_ at a highly significant level (*p* < 0.01).

In summary, total carotenoids content (TC_sp_) generally showed negative correlations with radical scavenging activities (DPPH) and reducing power (RP). Total phenols content (TP_sp_) in the water-rich phase (TP_wp_) showed strong positive correlations with radical scavenging activity (DPPH), indicating that higher phenolic content was associated with increased radical scavenging activity. Significant correlations highlight important interactions between these biochemical parameters, which could guide further research and optimization in cloud point extraction processes.

### 3.1. Principal Component Analysis

**Figure 2:**
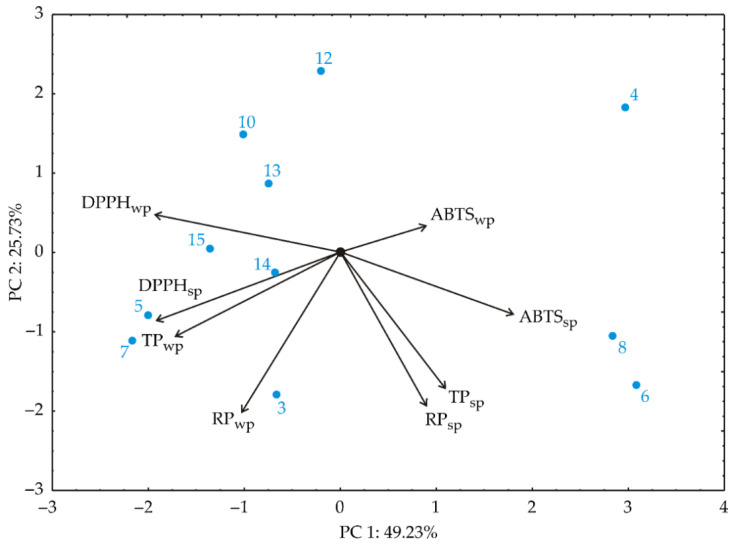
PCA ordination of variables based on component correlations is presented in the first and second factor planes, including total carotenoid content (TCs), total phenol content (TPs), DPPH radical scavenging activity, reducing power (RP), and ABTS radical scavenging activity (ABTS). “sp” in subscript indicates surfactant-rich phase, “wp” in subscript indicates water phase.

Initially, upon subjecting the collected experimental dataset to principal component analysis (PCA), distinct groupings among samples emerged based on the factor variables. Serving as an exploratory tool, this method facilitated the description and differentiation of response variables, as depicted in Figure 2. The PCA analysis indicated that the first two principal components explained 74.97% of the total variance, sufficient for data explanation. Importantly, specific factors notably influenced the first principal component (PC1). Variables such as TP_wp_, DPPH_sp_, and DPPH_wp_ contributed negatively to PC1 coordinate calculation: 16.93%, 21.17%, and 21.65%, respectively (based on correlations). Conversely, ABTS_sp_ had a positive influence on PC1, contributing 18.3% of the total variance. For the second principal component (PC2), factors such as TP_sp_, TP_wp_, RP_sp_, and RP_wp_ were more significant, negatively contributing 1.75%; 8.38%; 27.54%, and 29.92% of total variance, respectively, based on correlations. The PCA plot displays distinct groupings among the samples. Samples processed at higher temperature (run 4, 10, 12 and 13) are clearly positioned on the upper side of the chart. These samples exhibited higher levels of ABTS_wp_. In contrast, samples produced with lower pH value (runs 3, 5, and 7) demonstrated increased DPPH_sp_, RP_wp_, and TP_wp_. ABTS_sp_ and ABTS_wp_ had strong influences on PC 1, indicating their significant role in the variability of cloud point extraction outcomes. Consequently, enhancing conditions that increase ABTS_sp_ might decrease DPPH_sp_ due to their negative correlation. By understanding which parameters contribute most to variability, processes can be optimized to achieve desired outcomes, such as maximizing antioxidant activities (in view of ABTS) or phenolic content (TP_sp_).

### 3.2. Response Surface Method

The second-order polynomial (SOP) models were subjected to ANOVA analysis to investigate the effects of input variables, as detailed in Table 4. The results highlighted that in the SOP model for TC computation, the linear terms of pH had the most significant impact, demonstrating statistical significance at the *p* < 0.05 level. For the evaluation of TP_sp_, the SOP model indicated that the linear terms of temperature and time were significant at *p* < 0.01, and pH was significant at *p* < 0.05. The quadratic term of pH also significantly influenced TP_sp_ calculation at *p* < 0.01. Additionally, the interaction terms pH × Temp and pH × t were significant at *p* < 0.01 and *p* < 0.05, respectively. For TP_wp_ evaluation, the SOP model showed that the linear terms of pH and Temp were significant at *p* < 0.01, and t was significant at *p* < 0.05. The quadratic terms of pH and t were significant for TP_wp_ calculation at *p* < 0.05. Furthermore, the interaction terms pH × Temp and Temp × t were significant at *p* < 0.05. In the SOP model for DPPH_sp_ calculation, the linear term of pH was significant at *p* < 0.01. For RP_wp_ calculation, the linear term of pH was significant at *p* < 0.05. For DPPH_wp_ calculation, both the linear and quadratic terms of pH were significant at *p* < 0.05. Similarly, for ABTS_sp_ calculation, both the linear and quadratic terms of pH were significant at *p* < 0.05.

All SOP models successfully passed the lack-of-fit tests, confirming that they adequately represented the data. Furthermore, the high R^2^ values indicated a strong correlation between the model predictions and the experimental results.

**Figure 3:**
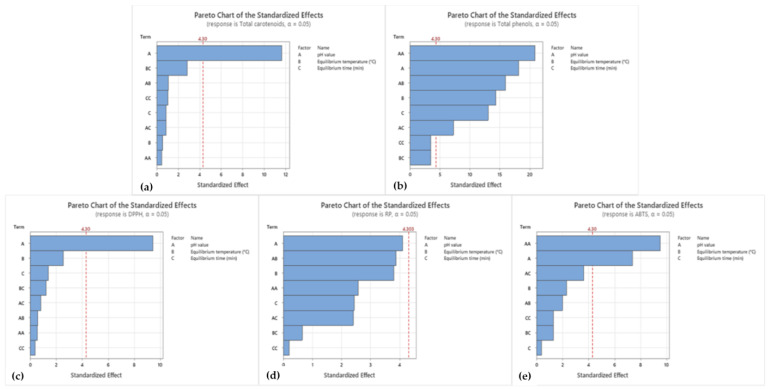
Pareto charts for CPE outcomes for surfactant-rich phase of CPE extract.

**Figure 4:**
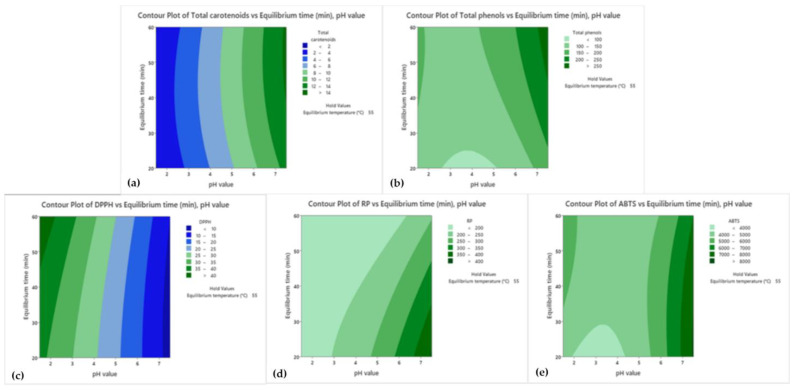
Contour plots for CPE outcomes for surfactant-rich phase of CPE extract.

For further correlations and improvements of operational CPE parameters (pH value, equilibrium temperature, and equilibrium time), Pareto charts and contour plots for surfactant-rich phase were created (Figure 3 and Figure 4). The same steps were repeated for the water phase (Supplementary Figures S1 and S2). The analysis of the Pareto charts for the surfactant-rich phase of CPE extract revealed significant insights into the factors affecting various responses, including total carotenoids, total phenols, DPPH, RP, and ABTS. For the response of total carotenoids, the pH value (Factor A) emerged as the most influential parameter, followed by equilibrium temperature (Factor B) and the interaction between pH value and equilibrium temperature (AB). This indicates that while pH value was critical, the combined effect of pH and temperature also played a substantial role in optimizing the total carotenoid content. Similarly, for total phenols, the pH value was again the predominant factor, with equilibrium temperature and equilibrium time (Factor C) also showing significant impacts. The interaction terms, especially AB (pH and temperature) and AC (pH and equilibrium time), highlight the importance of considering combined effects in optimizing phenol content. This suggests that a multi-faceted approach focusing on pH, temperature, and their interactions can yield better results. The DPPH response also followed a similar pattern, with pH value being the most critical factor, followed by equilibrium temperature and time. The interactions were less pronounced in this case, emphasizing the direct effects of these parameters. For the RP response, the trends were consistent, with pH value leading in significance, followed by temperature and time, with notable interactions between pH and temperature as well as pH and time. For the ABTS response, pH value remained a significant factor, with equilibrium temperature and their interaction (AB) also showing considerable effects. This underscores the necessity of optimizing both pH and temperature to achieve the best outcomes for ABTS.

From a broader perspective, these findings emphasize the principal importance of pH value across all measured responses. This suggests that controlling pH is essential in the CPE process, potentially more so than other parameters. The consistent significance of equilibrium temperature as the second most influential factor highlights its role in stabilizing the pH effects and ensuring optimal extraction conditions. The interactions between pH and temperature, evident in multiple responses, indicate that the optimization process cannot be isolated to single parameters. Instead, a proposed approach that considers the synergistic effects of multiple factors is necessary for achieving the best results.

As a step forward, the contour plots (Figure 4) for the surfactant-rich phase of CPE extract provide a visual representation of the effects of pH value and equilibrium time on various responses, including total carotenoids, total phenols, DPPH, RP, and ABTS. Namely, the contour plot for total carotenoids (Figure 4a) shows a clear gradient indicating the combined influence of pH value and equilibrium time. The highest levels of total carotenoids were observed at a pH value of around 7 and an equilibrium time of approximately 50 min. The plot suggests that higher pH values and longer equilibrium times are favorable for maximizing total carotenoid extraction. In terms of total phenols (Figure 4b), the highest content was achieved at a pH value of around 5, with equilibrium times extending up to 60 min. This indicates that total phenol extraction can be optimized at moderate pH levels and longer equilibrium times, slightly different from the conditions favorable for total carotenoids. The DPPH results (Figure 4c) demonstrate that the optimal pH value for maximizing antioxidant activity was around 3, with equilibrium times between 40 and 50 min. This suggests that lower pH values are more effective for enhancing DPPH activity, differing significantly from the optimal conditions for total carotenoids and phenols. Regarding the RP response (Figure 4d), the contour plot shows the highest reducing power at a pH value of around 5, with equilibrium times extending beyond 50 min. This is consistent with the trends observed for total phenols, indicating that similar conditions are favorable for both phenolic content and reducing power. The ABTS contour plot (Figure 4e) indicates that the highest antioxidant activity was achieved at a pH value near 3, with equilibrium times around 40 min. Similar to DPPH, lower pH values are more favorable for maximizing ABTS activity, highlighting the importance of acidic conditions for antioxidant responses.

Comparing these contour plots (Figure 4) with the Pareto charts (Figure 3) reveals consistent patterns where pH value plays a crucial role across all responses. However, the optimal pH varies depending on the specific outcome. For instance, total carotenoids are maximized at a higher pH, whereas antioxidant activities (DPPH and ABTS) are optimized at lower pH values. This indicates that different extraction outcomes require tailored conditions to achieve optimal results. Additionally, the equilibrium time also shows varying effects depending on the response. Longer equilibrium times tend to favor total carotenoids, total phenols, and RP, whereas shorter times are more suitable for DPPH and ABTS. This suggests that the kinetics of extraction processes differ for various phytochemicals and antioxidant activities.

### 3.3. Standard Score

**Figure 5:**
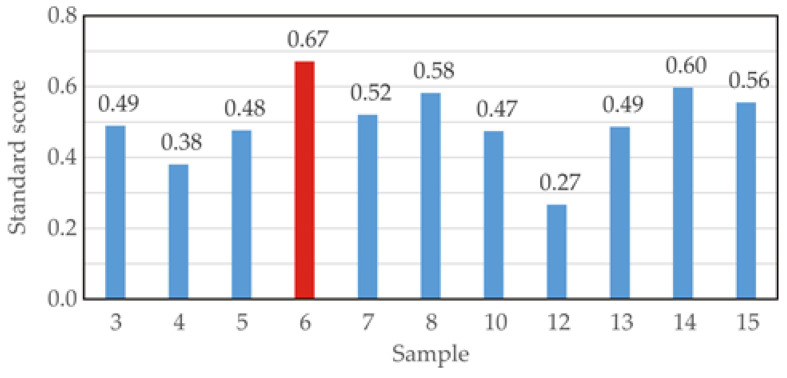
Standard scores.

These results of the standard scoring are illustrated in Figure 5. The SS analysis identified the most effective extraction conditions as those for sample No. 6, with a pH value of 7.5, equilibrium temperature of 55 °C, and equilibrium time of 20 min, resulting in high carotenoid and phenolic contents (TC_sp_ = 12.55 mg β-carotene/100 g, TP_sp_ = 180.02 mg GAE/100 g, TP_wp_ = 313.49 mg GAE/100 g). At these optimal extraction conditions, the antioxidant activity parameters of the sample were as follows: DPPH_sp_ = 8.3 μmol TE/100 g; DPPH_wp_ = 85.19 μmol TE/100 g; RP_sp_ = 406.26 μmol TE/100 g; RP_wp_ = 519.16 μmol TE/100 g; ABTS_sp_ = 8100.58 μmol TE/100 g; and ABTS_wp_ = 8900.48 μmol TE/100 g.

### 3.4. Verification of CPE Optimization Parameters and Assessment with Conventional Water Extraction

Based on the previously discussed results and outcomes, a response optimization was conducted using Box–Behnken design data to achieve maximum values for all CPE outcomes in the surfactant-rich phase. This optimization was performed with equal weight and importance coefficients for all outcomes, both set to 1. The rationale behind this approach was to obtain an extract with the best overall bioactivity. The optimized CPE operating parameters were calculated to be a pH value of 7.32, an equilibrium temperature of 55 °C, and an equilibrium time of 43.03 min. Using these optimized parameters, the predicted values for the CPE outcomes were as follows:

- Total carotenoids (TC_sp_): 13.80 mg β-carotene/100 g;
- Total phenols (TP_sp_): 236.14 mg GAE/100 g;
- DPPH (DPPH_sp_): 9.67 μmol TE/100 g;
- Reducing power (RP_sp_): 306.94 μmol TE/100 g;
- ABTS (ABTS_sp_): 7415.47 μmol TE/100 g.

To verify these predictive values, the CPE extraction was repeated using the optimized parameters. The experimental outcomes were then compared with the predicted values to assess the accuracy of the optimization (Table 5).

The verification of the CPE optimization revealed a high level of accuracy between the predicted and experimentally obtained values for all tested outputs. Briefly, the predictions for carotenoids were very close to the experimental results but slightly lower, with 13.80 and 13.11 mg β-carotene/100 g for predicted and obtained values, respectively. In contrast, the obtained values for phenols were higher than the predicted value of 3.57 mg GAE/100 g. Regarding the antioxidant potential, a slight overestimation in the predicted values for DPPH and ABTS was noticed, while reducing power exhibited slightly higher activity for 1.69 μmol TE/100 g.

To assess the efficacy of the CPE method, a comparative analysis was conducted involving conventional water extraction. It was found that optimized CPE showed superiority over conventional water extraction in terms of recovered bioactive compounds, particularly carotenoids. As expected, water extraction did not recover any carotenoids, due to their non-polar nature that limits their solubility in polar solvents like water. The efficacy of CPE in extracting carotenoids can be attributed to the use of Tween 80 as a surfactant, which possesses both polar and non-polar segments in its molecular structure. This amphiphilic nature of the surfactant allows it to form micelles in aqueous solutions, creating a hydrophobic environment that can effectively solubilize non-polar compounds like carotenoids. By incorporating carotenoids into these micelles, CPE enables their isolation from the plant material, resulting in significantly higher extraction yields compared with conventional water extraction. In addition, CPE also demonstrated superior performance in TP recovery and antioxidant activity, which suggests that the micellar formation and enhanced mass transfer facilitated by CPE can be beneficial for the extraction of a wide range of compounds from various sources. The studies by Motikar et al. [18] and Giovanoudis et al. [21] are also in line with these findings.

Matsusaka and Kawabata [42] demonstrated that extracting horned melon peel with 50% aqueous ethanol (*v*/*v*) at 30 °C for 24 h resulted in a polyphenol content of 5 mg/g. However, comparing our TP yields with those from other studies is challenging due to differences in horned melon cultivars and varying environmental conditions during growth, which significantly affect the types and amounts of compounds in the peel. The optimal extraction time of 43.03 min using CPE, compared with the 24 h duration employed [42], underscores the cost effectiveness of the CPE method. This approach not only shortens extraction time but also provides environmental and economic benefits by reducing solvent use, operating at lower temperatures, and minimizing energy consumption. These advantages align with sustainable extraction practices.

Recent research highlights a growing interest in closed-loop systems for reusing surfactants such as Tween 80, particularly for bioactive extracts obtained through CPE and their incorporation into various food products. This trend is driven by the dual aims of sustainability and cost effectiveness, along with the potential for technological advancements and wider applications in functional foods. For example, in yogurt production, where emulsifiers like Tween 80 are commonly used, there are promising technologies for enhancing yogurt with plant extracts rich in polyphenols and antioxidants [43,44]. Almeida et al. [45] suggested using Tween 80 for nano-encapsulation of curcumin, a polyphenol, as a dietary supplement in yogurt. Another strategy involves preparing plant extracts in surfactant solutions to create nanoemulsions that encapsulate polyphenols and other active compounds. These colloidal systems have shown benefits in drug delivery [46,47], and their application in food products could be highly advantageous. To fully integrate the extracts obtained using the proposed method in this study into industrial applications, additional research is necessary. Specifically, studies on the safety, microbiological aspects, and storage stability of the final food products are crucial.

### 3.5. Evaluation of Selected Food-Related Parameters

Microbiological food safety parameters are crucial for ensuring the safety and quality of plant extracts [21,48]. Therefore, the control horned melon peel extracts (obtained in water and optimized CPE extract) were assessed for key microbiological parameters (Table 6).

The total viable count for raw materials ranged from 2.5 to 3.5 log CFU, with the lyophilized horned melon peel showing the highest count at 3.5 log CFU/g. In comparison, the water-based extract maintained a similar value of 3.5 log CFU/mL, while the CPE extract demonstrated a significant reduction to 2 log CFU/mL, indicating the improved microbial safety of the CPE extract. For *Escherichia coli*, all raw materials and extracts tested below the detectable limit (<1 log CFU/mL or g), confirming no contamination by this pathogen. Similarly, *Salmonella* spp. and *Listeria monocytogenes* were not detected in any samples, affirming their safety regarding these harmful pathogens. The counts for *Staphylococcus aureus* were also below the detectable limit (<1 log CFU/mL or g) across all samples, suggesting good microbial quality. Yeasts and molds were higher in the water-based extract (2.5 log CFU/mL) compared with the raw materials but significantly lower in the CPE extract (1 log CFU/mL), highlighting the CPE process’s effectiveness in reducing fungal contamination. *Bacillus cereus* was detected at 1.8 log CFU/g in NaCl and 1.5 log CFU/mL in the water-based extract but was significantly reduced in the CPE extract to 0.5 log CFU/mL. This reduction indicates that the CPE process effectively minimized *Bacillus cereus* contamination. Enterobacteriaceae were below detectable limits (<1 log CFU/mL or g) in all samples, demonstrating a low risk of contamination from this family of bacteria. The CPE extract showed lower microbial values for key parameters, particularly total viable count, yeasts and molds, and *Bacillus cereus*, compared with the raw materials and water-based extract. This highlights the CPE process’s effectiveness in enhancing the microbiological safety and quality of horned melon peel extracts, making them suitable for food applications. The absence of harmful pathogens such as *Escherichia coli*, *Salmonella* spp., *Staphylococcus aureus*, and *Listeria monocytogenes* in all samples further emphasizes the safety of these extracts.

**Figure 6:**
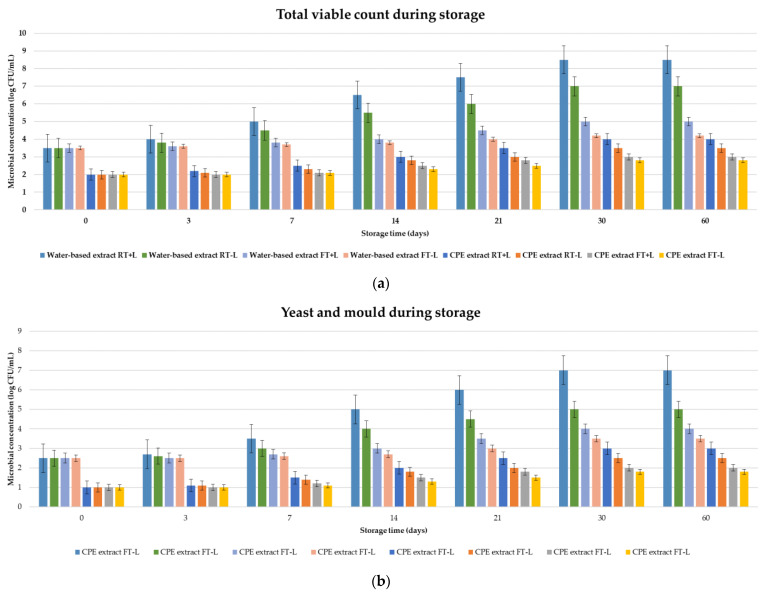
Storage stability of water-based and CPE extracts related to the microbiological parameters (room temperature with light (RT+L); room temperature without light (RT−L); refrigerated with light (FT+L); refrigerated without light (FT−L)): (**a**) total viable count; (**b**) yeast and mould count.

Additionally, the stability of water-based and CPE extracts of horned melon peel under different storage conditions (temperature and light exposure) was evaluated in order to gain valuable insights into their stability. The obtained results were compared with the initial values (defined as 100% value) and are presented in Figure 6. The total viable count as well as yeast and mold count were monitored over a period of 60 days, considering four different storage conditions: room temperature with light (RT+L), room temperature without light (RT−L), refrigerated with light (FT+L), and refrigerated without light (FT−L).

For the water-based extract, the total viable count increased substantially from 3.5 log CFU/mL at day 0 to 8.5 log CFU/mL by day 30 under RT+L conditions, indicating rapid microbial growth. Under RT−L, the total viable count showed a slightly slower increase, reaching 7.0 log CFU/mL by day 30. Storage under refrigerated conditions slowed microbial growth, with FT+L reaching 5.0 log CFU/mL and FT−L showing the slowest increase at 4.2 log CFU/mL by day 30. In contrast, the CPE extract demonstrated significantly better microbial stability. Under RT+L, the total viable count increased from 2.0 log CFU/mL at day 0 to 4.0 log CFU/mL by day 30. The RT−L conditions were associated with a slower increase to 3.5 log CFU/mL. Refrigerated conditions further improved stability, with FT+L reaching 3.0 log CFU/mL and FT−L showing the least growth at 2.8 log CFU/mL by day 30. The water-based extract’s yeast and mold counts also showed rapid increases under RT+L, rising from 2.5 log CFU/mL at day 0 to 7.0 log CFU/mL by day 30. The RT−L condition resulted in a slower increase to 5.0 log CFU/mL, while FT+L and FT−L conditions resulted in counts of 4.0 and 3.5 log CFU/mL, respectively, by day 30. For the CPE extract, yeast and mold counts under RT+L increased from 1.0 log CFU/mL at day 0 to 3.0 log CFU/mL by day 30. The RT−L conditions led to a slower increase to 2.5 log CFU/mL. Under refrigerated conditions, the counts were significantly lower, with FT+L reaching 2.0 log CFU/mL and FT−L showing the least growth at 1.8 log CFU/mL by day 30.

The data indicate that temperature is a significant factor affecting microbial growth. Room temperature conditions (RT) promoted higher microbial growth compared with refrigerated conditions (FT). Light exposure (+L) also contributed to increased microbial growth, suggesting that light may elevate temperatures or create a conducive environment for microbial proliferation. The combined effect of room temperature and light (RT+L) resulted in the highest microbial growth, while refrigerated and dark conditions (FT−L) provided the most inhibitory environment for microbial activity. The CPE extract consistently demonstrated superior microbial stability in comparison to the water-based extract. This stability can be attributed to the initial lower microbial load and the slower increase in microbial counts under all storage conditions. The cloud point extraction process appears to have been effective in reducing microbial contamination and enhancing the overall stability of the extract. Storing the extracts under refrigerated and dark conditions (FT−L) was particularly beneficial, as this combination significantly inhibited microbial growth. The CPE extract’s enhanced stability under these conditions makes it a more viable option for long-term storage, preserving both safety and quality.

## 4. Conclusions

The present paper sets out to a screening of the best-performing conditions, namely pH, equilibrium temperature, and equilibrium time, for cloud point extraction for the recovery of polyphenols and carotenoids from horned melon peel. Briefly, using improved parameters, i.e., pH value of 7.32, equilibrium temperature of 55 °C, and equilibrium time 43.03 min, it was possible to achieve higher recovery of selected bioactive compounds, resulting in 239.74 mg GAE/100 g and 13.11 mg β carotene/100 g of polyphenols and carotenoids, respectively. From the aspect of microbiological safety, the CPE extract demonstrated significantly lower microbial values for key parameters, including total viable count, yeasts and molds, and *Bacillus cereus*, compared with the raw materials and water-based extract. The CPE process appears to have been effective in reducing microbial contamination and enhancing the overall stability of the extract, making it a more suitable option for long-term storage and potential food applications. Specifically, storing the CPE extract under refrigeration without light conditions was particularly beneficial, as this combination significantly inhibited microbial growth, preserving both safety and quality under long-term storage. The findings not only demonstrate that CPE outperforms conventional extraction methods but also reveals new opportunities for the development of this highly efficient process of green extraction of both hydrophilic and hydrophobic compounds, allowing the extraction of bioactive compounds from complex matrices such as different by-products. This offers promising opportunities for the integration of valuable compounds obtained from byproducts into the food industry, as well as excellent waste-management solutions. However, to fully realize the potential of these extracts in functional foods, more advanced research is necessary.

## Figures and Tables

**Table 1:** Box–Behnken experimental design (BBD) with coded and actual levels of CPE parameters.

**Code**	**Variables**		**Coded levels**			
			−1	0		1
			**Actual levels**			
X_1_	pH value		1.5	4.5		7.5
X_2_	Equilibrium temperature (°C)		35	55		75
X_3_	Equilibrium time (min)		20	40		60
Experimental design						
Run	X_1_	X_2_			X_3_	
1	−1	−1			0	
2	1	−1			0	
3	−1	1			0	
4	1	1			0	
5	−1	0			−1	
6	1	0			−1	
7	−1	0			1	
8	1	0			1	
9	0	−1			−1	
10	0	1			−1	
11	0	−1			1	
12	0	1			1	
13	0	0			0	
14	0	0			0	
15	0	0			0	

**Table 2:** Box–Behnken-based experimentally observed responses (TC, TP, DPPH, RP, and ABTS) in CP extracts (surfactant-rich phase and water phase).

Run	Surfactant-Rich Phase (sp)				
	TC_sp_	TP_sp_	DPPH_sp_	RP_sp_	ABTS_sp_
	(mg β-Carotene/100 g)	(mg GAE/100 g)	(μmol TE/100 g)		
1	no phase separation occurred				
2	no phase separation occurred				
3	1.26 ± 0.50 ^a^	175.41 ± 3.33 ^f^	32.18 ± 2.42 ^f^	224.50 ± 20.41 ^de^	6360.79 ± 133.51 ^f^
4	14.65 ± 0.70 ^d^	129.91 ± 7.37 ^c^	4.90 ± 2.11 ^a^	137.07 ± 5.83 ^b^	7728.79 ± 164.56 ^h^
5	1.87 ± 0.44 ^a^	127.90 ± 7.35 ^c^	36.22 ± 1.15 ^g^	188.31 ± 23.23 ^d^	4253.30 ± 163.29 ^a^
6	12.55 ± 0.93 ^d^	180.02 ± 6.92 ^f^	8.30 ± 3.85 ^b^	406.26 ± 2.95 ^f^	8100.58 ± 116.45 ^g^
7	1.87 ± 0.71 ^a^	155.62 ± 3.48 ^e^	43.44 ± 3.38 ^h^	187.33 ± 11.65 ^d^	5659.71 ± 219.51 ^e^
8	14.29 ± 0.36 ^d^	277.34 ± 19.20 ^g^	10.16 ± 2.23 ^c^	244.08 ± 3.98 ^e^	6976.56 ± 450.90 ^fg^
9	no phase separation occurred				
10	9.02 ± 0.58 ^c^	62.33 ± 0.56 ^a^	21.68 ± 1.41 ^d^	144.01 ± 5.41 ^c^	4199.67 ± 56.38 ^a^
11	no phase separation occurred				
12	5.02 ± 0.72 ^b^	96.10 ± 3.34 ^b^	19.29 ± 3.23 ^d^	100.22 ± 8.57 ^a^	5125.92 ± 8.92 ^d^
13	6.90 ± 0.71 ^b^	127.30 ± 2.45 ^c^	21.30 ± 1.80 ^d^	176.25 ± 14.20 ^d^	4072.85 ± 84.77 ^a^
14	8.34 ± 0.43 ^c^	135.96 ± 2.78 ^d^	25.75 ± 2.72 ^e^	242.55 ± 9.84 ^e^	4770.06 ± 281.08 ^c^
15	8.80 ± 0.41 ^c^	135.18 ± 1.08 ^d^	27.61 ± 1.95 ^e^	200.66 ± 8.68 ^de^	4477.45 ± 41.27 ^b^
Run	Water phase (wp)				
	TP_wp_		DPPH_wp_	RP_wp_	ABTS_wp_
	(mg GAE/100 g)		(μmol TE/100 g)		
1	no phase separation occurred				
2	no phase separation occurred				
3	428.25 ± 33.90 ^e^		512.60 ± 8.53 ^e^	934.46 ± 73.75 ^g^	8752.68 ± 280.63 ^c^
4	198.61 ± 5.15 ^a^		120.39 ± 15.81 ^b^	179.23 ± 9.08 ^a^	8771.61 ± 439.02 ^c^
5	422.16 ± 17.24 ^e^		478.79 ± 14.91 ^d^	783.90 ± 17.19 ^f^	3521.47 ± 474.82 ^a^
6	313.49 ± 8.49 ^d^		85.19 ± 2.54 ^a^	519.16 ± 65.93 ^e^	8900.48 ± 847.70 ^cd^
7	461.62 ± 6.10 ^f^		770.68 ± 11.18 ^i^	737.75 ± 55.72 ^f^	6238.70 ± 342.31 ^b^
8	298.08 ± 4.27 ^c^		145.22 ± 7.93 ^c^	465.96 ± 21.82 ^d^	10,080.85 ± 208.80 ^d^
9	no phase separation occurred				
10	395.27 ± 7.96 ^e^		636.58 ± 39.80 ^gh^	519.75 ± 32.39 ^e^	11,359.71 ± 388.24 ^d^
11	no phase separation occurred				
12	272.07 ± 4.45 ^b^		658.28 ± 3.36 ^gh^	240.21 ± 27.70 ^b^	6907.59 ± 736.16 ^b^
13	437.80 ± 11.59 ^e^		548.64 ± 1.80 ^f^	342.82 ± 25.42 ^c^	8572.07 ± 159.05 ^c^
14	452.62 ± 6.38 ^f^		600.20 ± 9.76 ^g^	546.86 ± 33.65 ^e^	10,654.54 ± 335.51 ^d^
15	428.25 ± 33.90 ^e^		512.60 ± 8.53 ^e^	934.46 ± 73.75 ^g^	8752.68 ± 280.63 ^c^

Legend: total carotenoids content (TCs), total phenols content (TPs), radical scavenging activity (DPPH), reducing power (RP), and ABTS radical scavenging activity (ABTS). Different letters in the table columns indicate that the mean values are statistically different at the *p* < 0.05 level, according to Tukey’s HSD test.

**Table 3:** Correlation matrix of the cloud point extraction parameters (“sp” in subscript marks surfactant-rich phase, “wp” in subscript marks water phase).

Responses	TP_sp_	TP_wp_	DPPH_sp_	DPPH_wp_	RP_sp_	RP_wp_	ABTS_sp_	ABTS_wp_
TC_sp_	0.300	−0.633 *	−0.896 ***	−0.723 *	0.276	−0.662 *	0.526	0.587
TP_sp_		−0.146	−0.216	−0.557	0.552	0.202	0.583	0.100
TP_wp_			0.818 **	0.704 *	0.078	0.662 *	−0.670 *	−0.153
DPPH_sp_				0.790 **	−0.211	0.732 *	−0.591	−0.524
DPPH_wp_					−0.506	0.278	−0.808 **	−0.227
RP_sp_						0.307	0.510	0.184
RP_wp_							−0.126	−0.269
ABTS_sp_								0.205

*** Statistically significant at *p* < 0.001 level, ** statistically significant at *p* < 0.01 level, * statistically significant at *p* < 0.05 level. Total carotenoids content (TC), total phenols content (TP), radical scavenging activity (DPPH), reducing power (RP), and ABTS radical scavenging activity (ABTS).

**Table 4:** ANOVA evaluation of parameters for cloud point extraction process (“sp” in subscript marks surfactant-rich phase, “wp” in subscript marks water phase).

Terms	df	TC_sp_	TP_sp_	TP_wp_	DPPH_sp_	DPPH_wp_	RP_sp_	RP_wp_	ABTS_sp_	ABTS_wp_
pH	1	2.1 × 10^2^ **	5.7 × 10^2^ *	4.5 × 10^4^ **	1.1 × 10^3^ **	2.7 × 10^5^ *	8.3 × 10^2^	3.5 × 10^5^ *	5.2 × 10^6^ *	7.1 × 10^6^
Temp	1	0.3	4.7 × 10^3^ **	1.9 × 10^4^ **	6.8	1.0 × 10^2^	1.6 × 10^4^	1.5 × 10^4^	6.6 × 10^5^	2.1 × 10^6^
t	1	3.3	3.1 × 10^3^ **	4.1 × 10^3^ *	1.5	1.3 × 10^4^	5.2 × 10^3^	3.6 × 10^4^	3.8 × 10^5^	2.1 × 10^6^
pH^2^	1	0.2	1.0 × 10^4^ **	5.7 × 10^3^ *	3.3	2.0 × 10^5^ *	7.5 × 10^3^	7.4 × 10^4^	1.1 × 10^7^ *	2.7 × 10^6^
t^2^	1	1.1	2.8 × 10^2^	1.9 × 10^3^ *	1.6	5.6 × 10^3^	4.7 × 10^1^	9.6 × 10^1^	2.1 × 10^5^	1.1 × 10^6^
pH × Temp	1	1.1	5.8 × 10^3^ **	2.9 × 10^3^ *	3.7	4.6 × 10^3^	1.7 × 10^4^	7.9 × 10^4^	4.9 × 10^5^	7.0 × 10^6^
pH × t	1	0.8	1.2 × 10^3^ *	7.5 × 10^2^	7.2	1.3 × 10^4^	6.5 × 10^3^	1.2 × 10^1^	1.6 × 10^6^	5.9 × 10^5^
Temp × t	1	7.9	2.8 × 10^2^	6.1 × 10^3^ *	1.6 × 10^1^	7.9 × 10^3^	4.8 × 10^2^	1.8 × 10^4^	2.1 × 10^5^	1.4 × 10^7^
Error	2	2.0 × 10	4.6 × 10^1^	1.8 × 10^2^	2.1 × 10^1^	7.6 × 10^3^	2.2 × 10^3^	2.3 × 10^4^	2.5 × 10^5^	5.4 × 10^6^
R^2^		0.992	0.998	0.998	0.985	0.987	0.965	0.956	0.989	0.889
adj R^2^		0.958	0.992	0.989	0.927	0.936	0.826	0.782	0.943	0.444

** statistically significant at *p* < 0.01 level, * statistically significant at *p* < 0.05 level. Legend: Temp—equilibrium temperature; t—equilibrium time; total carotenoids content (TC), total phenols content (TP), radical scavenging activity (DPPH), reducing power (RP), and ABTS radical scavenging activity (ABTS).

**Table 5:** Experimental verification of predicted CPE outcomes values using optimized CPE parameters and comparison with conventional water extraction.

CPE Optimization			
CPE phase:		Surfactant-rich phase	
Goal for CPE outcomes values:		Maximizing	
Weight coefficient for CPE outcomes:		equal for all (1)	
Importance of CPE outcomes:		equal for all (1)	
Optimized CPE parameters			
pH value (/)		7.32	
Equilibrium temperature (°C)		55	
Equilibrium time (min)		43.03	
CPE outcomes for optimized CPE parameters			
Predicted CPE outcomes		Experimentally obtained CPE outcomes	
TC (mg β carotene/100 g)	13.80	TC (mg β-carotene/100 g)	13.11
TP (mg GAE/100 g)	236.14	TP (mg GAE/100 g)	239.71
DPPH (μmol TE/100 g)	9.67	DPPH (μmol TE/100 g)	9.05
RP (μmol TE/100 g)	306.94	RP (μmol TE/100 g)	308.63
ABTS (μmol TE/100 g)	7415.47	ABTS (μmol TE/100 g)	7398.58
Comparison with conventional water extraction			
TC (mg β carotene/100 g)	nd *		
TP (mg GAE/100 g)	165.21 ± 0.21		
DPPH (μmol TE/100 g)	117.78 ± 0.36		
RP (μmol TE/100 g)	156.28 ± 1.47		
ABTS (μmol TE/100 g)	3274.02 ± 5.16		

* Not detected.

**Table 6:** Microbiological food safety parameters for raw materials and final extracts.

Microbiological Parameter (log CFU/mL or g)	Raw Materials				Obtained Extracts	
	Water	NaCl	Tween 80	Lyophilized Horned Melon Peel	Water-Based Extract	CPE Extract
Total viable count	3	2.5	2.8	3.5	3.5	2
*Escherichia coli*	<1	<1	<1	<1	<1	<1
*Salmonella* spp.	nd	nd	nd	nd	nd	nd
*Staphylococcus aureus*	<1	<1	<1	<1	<1	<1
*Listeria monocytogenes*	nd	nd	nd	nd	nd	nd
Yeast and mold	0.3	0.8	1.1	1.4	2.5	1
*Bacillus cereus*	<1	1.8	<1	0.4	1.5	0.5
Enterobacteriaceae	<1	<1	<1	<1	<1	<1

## Data Availability

The original contributions presented in the study are included in the article/supplementary material, further inquiries can be directed to the corresponding author.

## Supplementary Materials

The following supporting information can be downloaded at https://www.mdpi.com/article/10.3390/foods13182863/s1, Figure S1: Pareto charts for CPE outcomes for water phase of CPE extract; Figure S2. Contour plots for CPE outcomes for water phase of CPE extract.
