# Supplementary material for: Sustainable Recovery of Polyphenols and Carotenoids from Horned Melon Peel via Cloud Point Extraction
Source: Foods. 2024 Sep 10;13(18):2863. doi: 10.3390/foods13182863 (PMC11431220; doi:10.3390/foods13182863)
Supplement: Supplementary file 1 [file foods-13-02863-s001.zip › foods-3196230-supplementary.pdf]

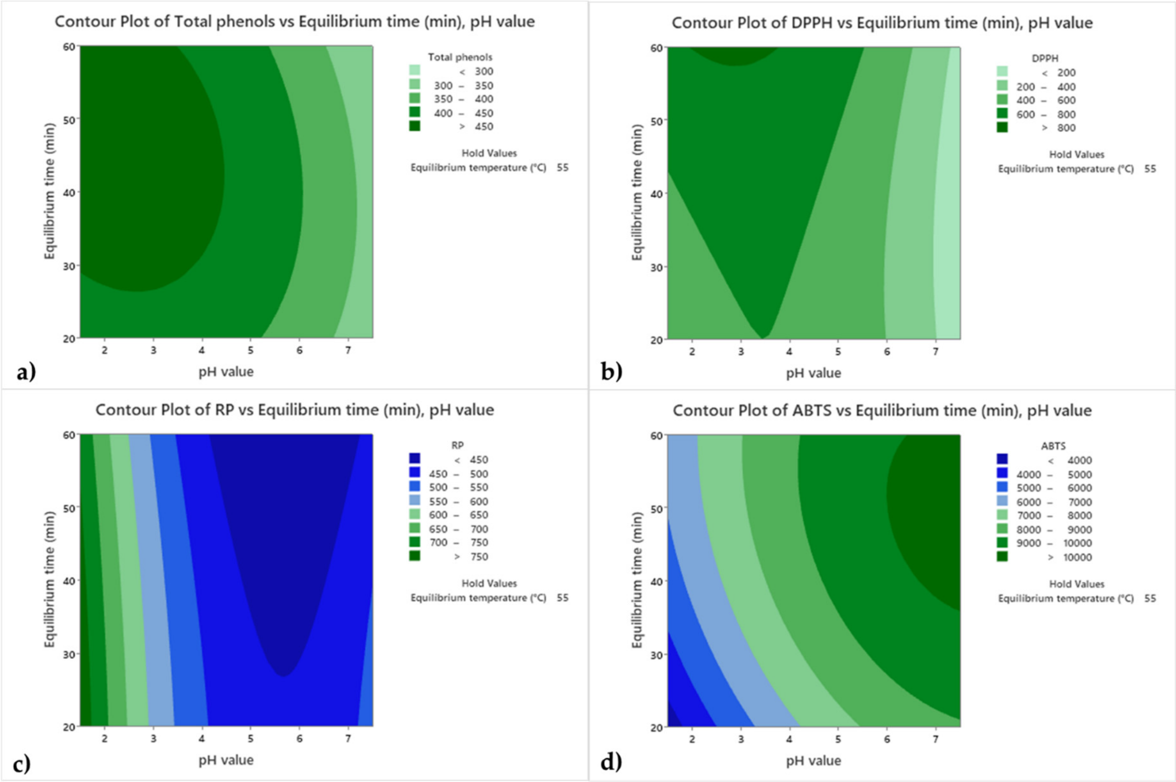

Figure S1: Pareto charts for CPE outcomes for water phase of CPE extract

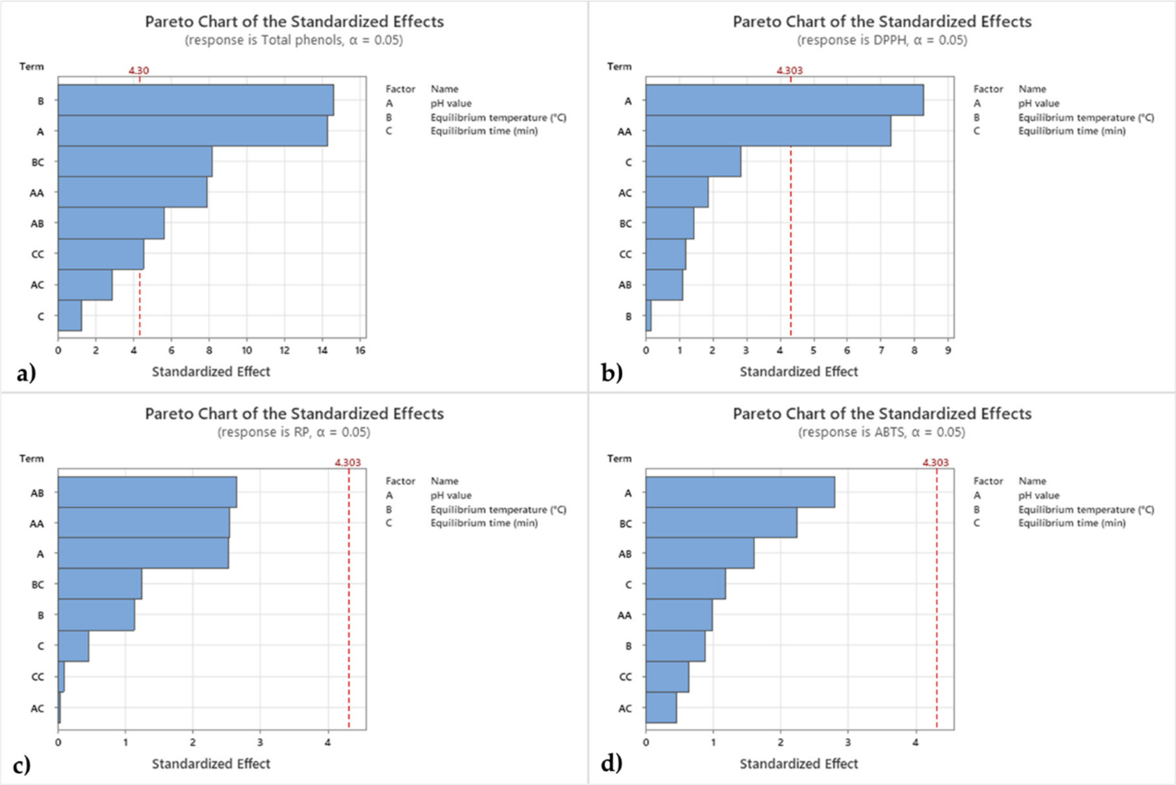

Figure S2. Contour plots for CPE outcomes for water phase of CPE extract
